# The Influence of the Acceleration Admixture Type and Composition of Cement on Hydration Heat and Setting Time of Slag Blended Cement

**DOI:** 10.3390/ma15082797

**Published:** 2022-04-11

**Authors:** Jan Pizoń, Beata Łaźniewska-Piekarczyk, Patrycja Miera

**Affiliations:** Faculty of Civil Engineering, Silesian University of Technology, 44-100 Gliwice, Poland; beata.lazniewska-piekarczyk@polsl.pl (B.Ł.-P.); patrycja.miera@polsl.pl (P.M.)

**Keywords:** cement, slag blended cement, accelerating admixtures, concrete, hydration heat, initial setting time, crystal seeds, calcium nitrate, cement kiln dust, sodium hydroxide

## Abstract

This article presents recent research on cements containing GGBFS and their modifications with accelerating admixtures. The initial setting time and hydration heat evolution results are presented for cement CEM II/B-S and CEM III/A manufactured with three Portland clinkers of various phase compositions. The research was carried out at 8 °C and 20 °C. The main objective is to assess the behavior of blended cements in cooperation with modern admixtures that contain nucleation seeds. The authors aimed to compare and evaluate different methods to reduce setting time, namely, the effects of temperature, the specific surface area of cement and GGBFS, the type of Portland clinker, the content of GGBFS, and presence of accelerators. Many of these aspects appear in separate studies, and the authors wanted a more comprehensive coverage of the subject. Those methods of reducing the setting time can be ranked: the most effective is to increase the temperature of the ingredients and the surroundings, the second is to reduce the GGBFS content in cement, and the use of accelerators, and the least effective is the additional milling of Portland clinker. However, of these methods, only the use of accelerators is acceptable in terms of sustainability. Prospective research is a detailed study on the amounts of C-S-H phase and portlandite to determine the hydration rate.

## 1. Introduction

Concrete is one of the most popular building materials. Since the patenting of Portland cement, increasing technical and environmental requirements have forced modifications to this material. One of the first modifiers used was accelerating admixtures. In 1885 the first compound for this purpose, calcium chloride, was used [1]. Initially, accelerating admixtures were used due to the lower specific surface area of cement and to concrete at reduced temperatures [1,2]. Today, they are also used in precast production and to reduce formwork rental time [3,4]. However, calcium chloride was soon found to cause steel corrosion, necessitating the invention of other compounds to replace it. However, the use of calcium chloride continues to be investigated, including protecting and repairing objects [5,6]. Accelerating admixtures contain the following components: nitrates and nitrites, formates; carbonates; diethanolamine (DEA); triethanolamine (TEA); triisopropanolamine (TIPA); bromides; fluorides; alkali hydroxides [3,4,7,8,9,10,11]. Currently, nanomaterial-containing admixtures are developing more rapidly. They are referred to as C-S-H seeds, crystal seeds, nanosize C-S-H, nanocrystals, nucleation seeds, nano modifiers, and other names. New developments are still being sought [12,13,14,15,16].

A leading trend to mitigate the negative environmental impact of cement production is the use of waste materials [17,18]. Due to the widespread use of ground granulated blast furnace slag (GGBFS), cement containing it was the core material used in this study. The influence of slag on the heat of hydration is described by many authors [19,20,21]. These sources indicate that GGBFS reduces the amount of hydration heat released and the intensity of hydration heat evolution. Some also indicate the appearance of the characteristic second peak on the heat release graph caused by the GGBFS reaction for several slag cements [22,23]. Furthermore, the initial setting time is delayed [24,25], although some sources present the beneficial effect of a small amount of slag to shorten it; this may be attributed to an effect similar to crystal seeds [26].

The effect of the specific surface area of GGBFS on the initial setting time and the amount of hydration heat released is also reported. The hydration reaction rate increases as the fineness of the slag increases. This is related to the larger surface area available for hydration reaction to occur [23,27]. The specific surface area of cement has a similar effect but is more significant than that of slag due to the higher reactivity at the beginning of the hydration process [28,29,30]. Although the influence of specific surface area is well known, it should be assessed to determine whether it is possible to override it with accelerators.

The phase composition of Portland clinker varies according to the feed of the raw materials and methods used in its production. Clinker is a composition of tricalcium silicate (alite—C_3_S), dicalcium silicate (belite—C_2_S), tricalcium aluminate (celite—C_3_A), and tetracalcium aluminate (brownmillerite—C_4_AF). Phase composition ranges for C_3_S are: 45–70%, C_2_S: 10–30%. C_3_A: 5–12%, C_4_AF: 5–12% [31,32]. The heat of hydration released by the individual phases is shaped as follows: C_3_S 500 J/g, C_2_S 260 J/g C_3_A 870 J/g, C_4_AF 420 J/g [2]. C_3_S has the greatest share in shaping the heat evolution of hydration due to its high heat release rate and its highest content, and C_3_A due to its highest heat release rate. In the initial hydration stage, C_3_A has the greatest influence due to the fastest reaction [32,33]. There are few comprehensive comparisons of the hydration heat and setting time with different phase compositions. Single test results are reported for specific cements. Cement containing more C_3_A achieved the highest amount of heat of hydration released. The highest heat release does not always coincide with the fastest initial setting time [34].

As the ambient temperature increases, the rate of chemical reactions increases. The effect of increasing the temperature during the hydration reaction and its effect on the heat release and initial setting time is better described in the literature. Effects are described for Portland cement [35,36] and cement containing non-clinker components [37,38]. However, comparisons of increased temperature and other factors (specific surface area, clinker type, accelerators, taken into account in the present research) affecting the acceleration of the hydration reaction are lacking.

Admixtures containing C-S-H seeds are currently the subject of considerable research. Most of them report beneficial effects on the properties of Portland cement [39,40,41,42]. Fewer sources show the interaction of these admixtures with blended cement. Cements containing fly ash [43,44] and GGBFS [45,46] were the subject of such research. Improved mechanical properties, reduced initial setting time, and effect on consistency and durability of cement composites are described. In most cases, there is a beneficial effect. 

Calcium nitrate is a well-studied concrete accelerating admixture. Its performance is well documented at average and reduced temperatures and in cooperation with Portland cement and blended cement. Beneficial effects on mechanical properties and initial setting time are described [7,10,47,48]. However, the possibility of calcium nitrate overdose should be considered, which results in adverse effects [7,11]. In the current study, this admixture was used to compare the effectiveness of the others.

Cement kiln dust (CKD) is a waste generated during cement production in the rotary kiln [49,50,51] and is considered a hazardous waste [52]. The effect of CKD on concrete properties varies and depends on the raw materials used in cement production, the type of production technology, and the dust capture installation [49]. Currently, CKD is used in soil stabilization, low-strength concrete manufacture, asphalt concrete production, or artificial aggregate preparation [53,54]. Furthermore, CKD is useful in the preparation of alkali-activated blast furnace slag binders (AAS) [52]. Therefore, they may help accelerate the hydration of cement containing GGBFS. The effect of accelerating the initial setting time and improving mechanical properties has been reported [55,56]. CKD is also used in collaboration with cement containing fly ash, GGBFS, and silica dust [57,58]. However, massive amounts of added CKD can degrade composite properties [58,59]. Therefore, CKDs have been involved in research with contradictory reports on their effectiveness and performance.

Sodium hydroxide, which, when dissolved in water, increases its pH, may have a similar activating effect on the latent hydraulic properties of GGBFS [60,61]. It is also used in AAS [61,62]. The beneficial effect of sodium hydroxide can only be observed during the initial hydration period. After a longer period, the strength properties deteriorate [63,64]. Sodium hydroxide is also used to activate fly ash cement [65,66].

The motivation for this study was the growing popularity of modern admixtures to accelerate setting and hardening containing nucleation seeds. C-S-H seed admixtures should be compared with other methods; this is evident in the number of recent articles published on the subject. The main objective is to assess the behavior of blended cements in cooperation with this admixture because little comprehensive research for GGBFS blended cement has been reported.

The authors wanted to address mainly cement containing GGBFS in order to compare different methods of reducing the setting time: the effects of temperature, the specific surface area of cement and GGBFS, the type of Portland clinker, the content of GGBFS, and presence of accelerators. Many of these aspects appear in separate studies, and the authors wanted a deeper coverage of the subject. This comparison is the best value of this article.

In previous work, the authors have addressed the topics of activators in terms of the microstructure of hardened cement paste, strength, and initial setting time [64,67,68]. Prospective research is a detailed study on amounts of C-S-H phase and portlandite to determine the hydration rate. XRD and TGA-DTG methods for different ages of blended cement pastes may be used.

## 2. Materials and Methods

### 2.1. Cement

**Figure 1:**
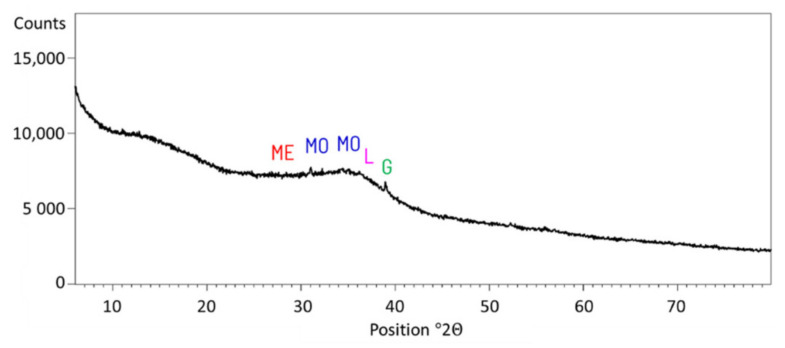
XRD analysis of GGBFS. ME—Merwinite; MO—Monticellite; L—Larnite; G—Gelhenite.

**Figure 2:**
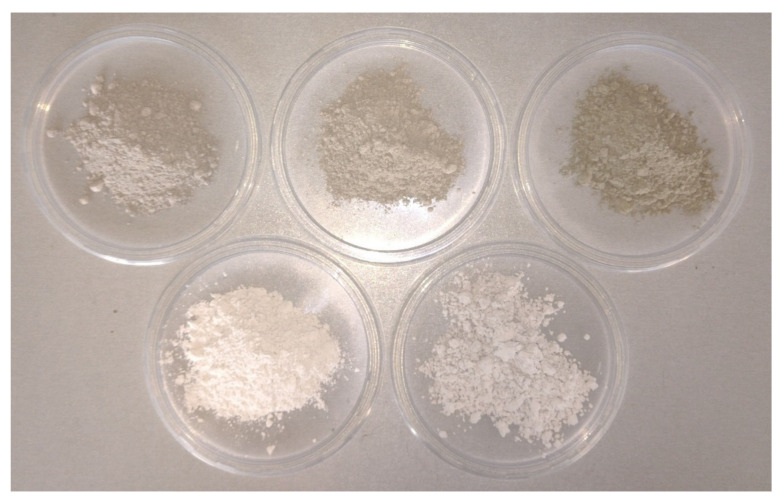
Clinkers (top row, from left C1, C2, C3), GGBFS (bottom row left), and anhydrite (bottom row right) involved in the research.

**Figure 3:**
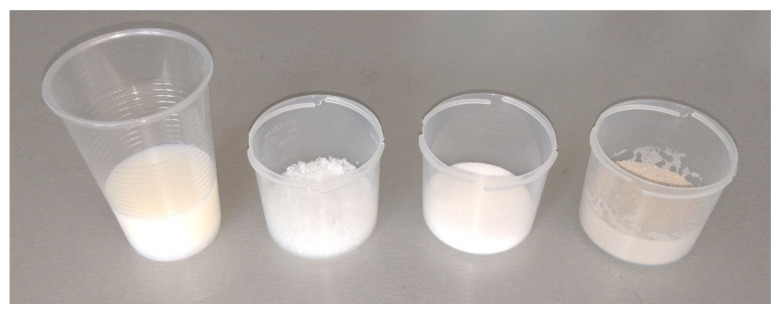
Accelerators involved in the research, from left: crystal seeds admixture, calcium nitrate, sodium hydroxide, CKD.

The cement used in the research was manufactured in the laboratory from Portland clinker, ground granulated blast-furnace slag (GGBFS), and anhydrite (set regulator). Portland clinker was replaced with GGBFS in amounts of 0% (equivalent for CEM I), 35% (equivalent for CEM II/B-S), and 65% (equivalent for CEM III/A). The properties of GGBFS exceed the minimum values specified by the standards. GGBFS reactivity indices are greater than required by EN 15167—1: 62.8% after seven days (against 45% required) and 88.3% after 28 days (compared to 70% required). The amorphous phase content of the slag is 98.5%, which is also greater than required by EN 197—1 67%. The chemical characteristics and specific surfaces of the GGBFS used are given in Table 1. The GGBFS XRD analysis is presented in Figure 1. Three different Portland clinkers were used in the investigation. Clinkers differed in their phase composition. Every clinker was ground to Blaine’s specific surface of 3000 cm^2^/g, 4000 cm^2^/g, and 5000 cm^2^/g. The phase composition, determined by the Bogue equations, of the Portland clinkers is given in Table 2. Their chemical composition is presented in Table 3. Anhydrite amounts were established for each blend separately to obtain 2% SO_3_ in the cement. The characteristics are presented in Table 4. All materials involved in the research are presented in Figure 2 (clinkers and GGBFS) and Figure 3 (accelerators).

### 2.2. Accelerators

Four different accelerators were used in the research.

**Figure 4:**
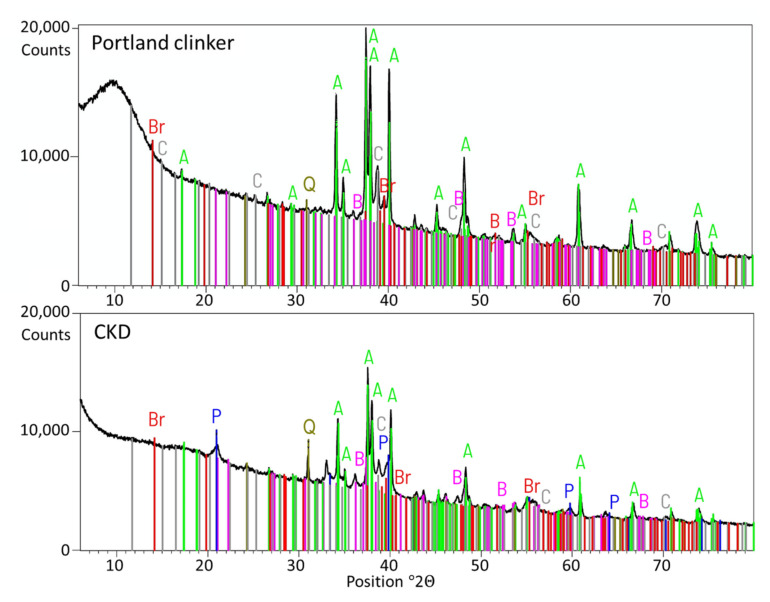
XRD analysis of CKD and the Portland clinker from which production it was obtained. A—tricalcium silicate; B—dicalcium silicate; C—tricalcium aluminate; Br—tetracalcium aluminoferrite; Q—quartz; P—portlandite.

**Figure 5:**
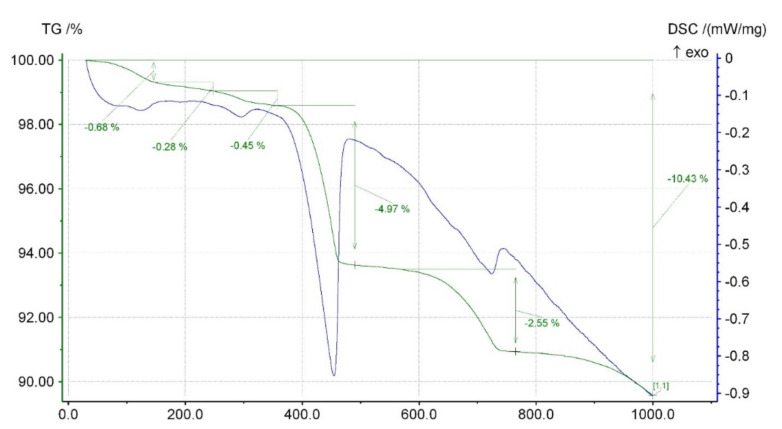
TG-DCS analysis of CKD.

1. A modern set and hardening acceleration admixture (symbol S) is available on the market. It contains crystal seeds in the form of C-S-H nanoparticles.
2. Traditional accelerating admixture—calcium nitrate (symbol C). The 20% solution of calcium nitrate tetrahydrate (Ca(NO_3_)_2_·4H_2_O) was added to reach the amount of 2% calcium nitrate per cement mass.
3. The 20% sodium hydroxide solution (NaOH; symbol N) was added to reach the amount of 5% sodium hydroxide per cement mass.
4. Cement kiln dust (CKD) (symbol D) was obtained from one of the polish cement plants. CKD was introduced as an ingredient in cement in the amount of 10% of the total mass of cement. The chemical composition of CKD is given in Table 5. CKD XRD and TG-DCS analyses are presented in Figure 4 and Figure 5. The resemblance of the chemical composition described before is visible.

### 2.3. Mixtures

Cement pastes were used as research materials. These were mixed using the ingredients described above. The compositions of the pastes for the initial setting time tests are given in Table 6. The amounts of water were established while obtaining the standard consistency according to EN 196-3. The results of the water demand are given in Table 7. Hydration heat tests were conducted on a 5 g cement sample with a water–cement ratio of 0.5 and a proportional amount of accelerators, according to Table 6.

### 2.4. Methods

Standard consistency and initial setting time of cement were tested according to procedures given in the standard EN 196-3. The tests were carried out using an automated Vicat apparatus, manufactured by Matest, Arcore, Italy.

Hydration heat tests were conducted using an isothermal calorimeter TAM Air. The device uses stirring ampoules that allow heat measurement from the moment of water and cement contact. The tests were continuous for 72 h; the results were recorded each minute. 

All components were stored at the appropriate temperature (8 °C or 20 °C) for at least 24 h before testing. The mixtures were prepared in the room at a stable temperature. Finally, the test equipment was placed in a room with a suitable temperature.

## 3. Results

### 3.1. Initial Setting Time

**Figure 6:**
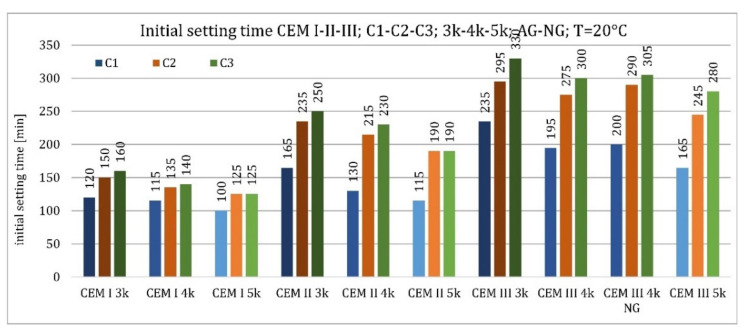
Initial setting time of non-modified cements CEM I, CEM II/B-S, CEM III/A at 20 °C.

The influence of clinker type on the initial setting time of the unmodified pastes at 20 °C is shown in Figure 6. Cements made from clinker C1, which contained 13.5% C_3_A, were characterized by the shortest initial setting time. The cements made of the other two clinkers (containing 4.0% and 2.4% C_3_A) exhibited longer initial setting times. These relationships were true when Portland cement CEM I, Portland slag cement CEM II/B-S, and blast furnace CEM III/A cement were tested at 20 °C, and CEM I cements at 8 °C. More significant relative increases in differences were observed for cement containing GGBFS.

**Figure 7:**
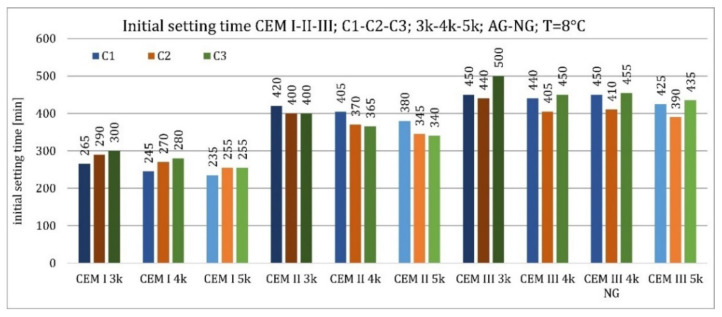
Initial setting time of non-modified cements CEM I, CEM II/B-S, CEM III/A at 8 °C.

The initial setting time increased with increasing GGBFS amount in cement and decreasing ambient temperature and specific surface area (Figure 6 and Figure 7). More considerable relative differences were observed at 20 °C. This was a general decrease in the hydration reaction rate at the reduced temperature. The specific surface area of ground granulated blast furnace slag did not affect the initial setting time due to its near-neutral nature at the initial stage of cement pastes curing (Figure 6 and Figure 7).

The initial setting time was less variable at lower temperatures (Figure 7). The relationships for GGFBS content and clinker-specific surface area corresponded to those for the higher temperature. The clinker type affected the same for Portland cement CEM I but differently for the other two types of cement. For CEM II/B-S, clinkers with a lower C_3_A content (C2—4.0% and C3—2.4%) exhibited shorter setting times than cement made from C1 (13.5% C_3_A). For CEM III/A cement, clinkers C1 and C2 showed shorter initial setting times than clinker C3.

**Figure 8:**
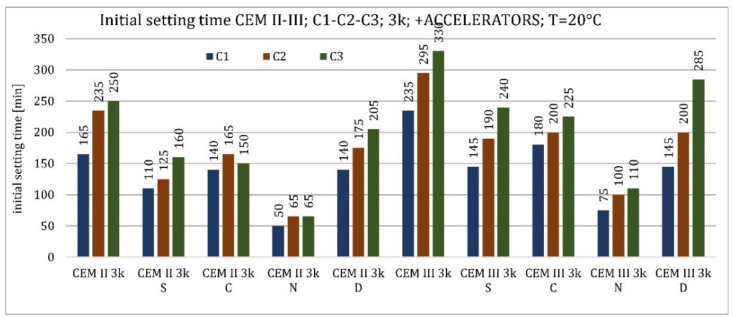
Initial setting time of cements CEM I, CEM II/B-S, CEM III/A modified with accelerators at 20 °C.

**Figure 9:**
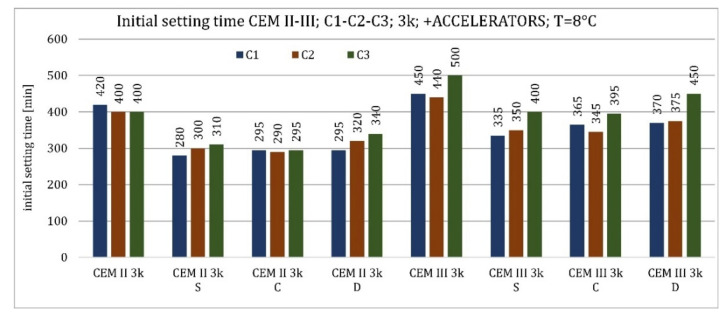
Initial setting time of cements CEM I, CEM II/B-S, CEM III/A modified with accelerators at 8 °C.

The results of the accelerator-modified cement tests are presented in Figure 8 and Figure 9. All activators caused a reduction in the initial setting time. For all cements, the most significant effect in reducing the initial setting time occurred with sodium hydroxide (reducing the initial setting time by approximately 300–400% depending on the type of cement). The next most effective accelerator was an admixture containing nucleation seeds for CEM II/B-S and CEM III/A cements made from C1 and C2 clinkers, as well as for CEM II/B-S cement made from C3 clinker (reduction of approximately 50–100%). In the case of CEM III/A cement made of C3, calcium nitrate replaced it. The least effective was to accelerate the initial setting time with CKD (reduction in the initial setting time by approximately 10–50%). Only in the case of CEM III/A cement made of C1 clinker did CKDs reduce the setting time more than calcium nitrate.

In the case of cements modified with setting activators, the relationships followed a pattern similar to that at higher temperatures. All the activators used reduced the initial setting time. In cement made with clinker C1, the most effective activator was nucleation seeds, and the second most effective was calcium nitrate. In cements made with the other two clinkers, the relationship reversed and the beneficial effect of a higher C_2_S content in cement containing C3 clinker, in cooperation with calcium nitrate, was more pronounced than at higher temperature. CKDs reduced the initial setting time the least. The effect of sodium hydroxide was not studied at lower temperatures (Figure 8 and Figure 9).

### 3.2. Hydration Heat Evolution

**Figure 10:**
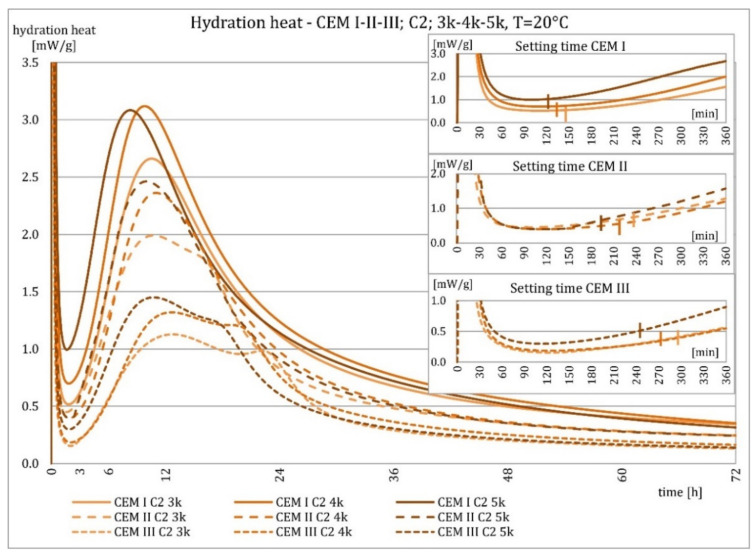
Hydration heat of C2 clinker based non-modified cements CEM I, CEM II/B-S, CEM III/A at 20 °C.

**Figure 11:**
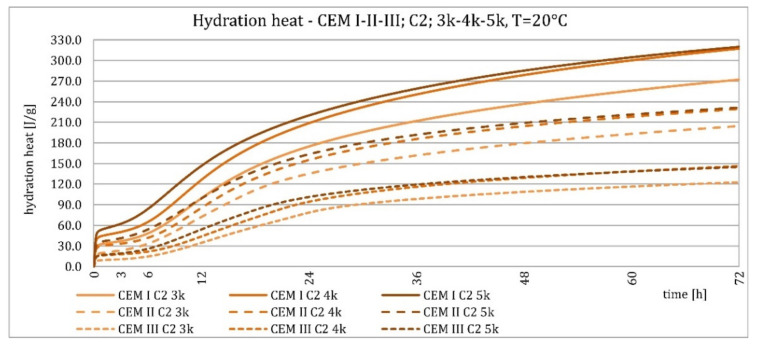
Hydration heat of C2 clinker based non-modified cements CEM I, CEM II/B-S, CEM III/A at 20 °C.

**Figure 12:**
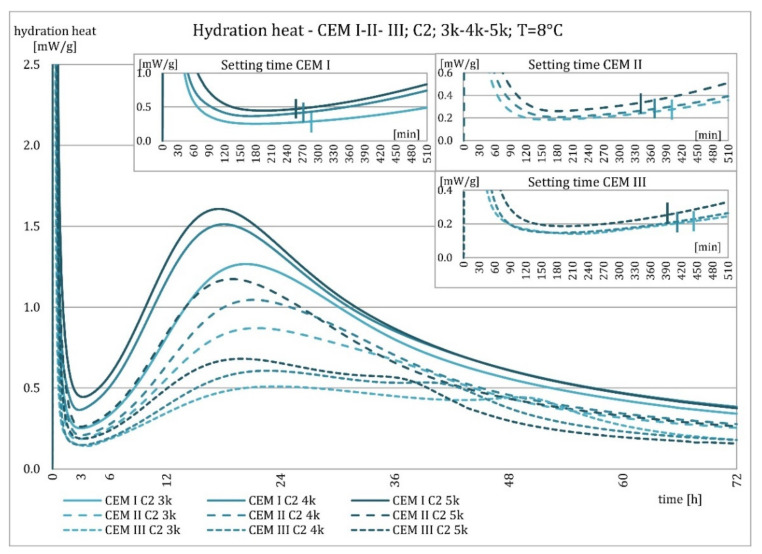
Hydration heat of C2 clinker based non-modified cements CEM I, CEM II/B-S, CEM III/A at 8 °C.

**Figure 13:**
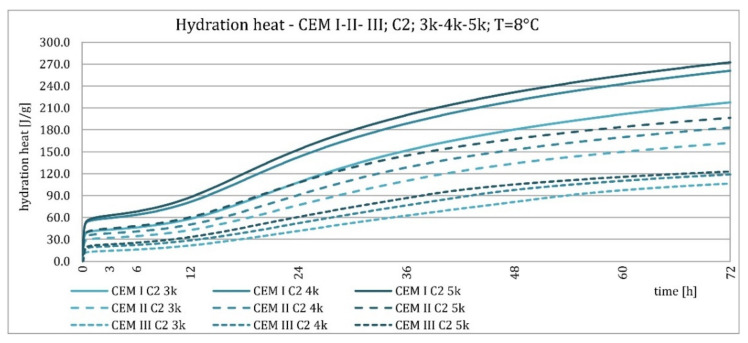
Hydration heat of C2 clinker based non-modified cements CEM I, CEM II/B-S, CEM III/A at 8 °C.

The results of the evolution of the hydration heat of unmodified cements made from C2 clinker with specific surface areas of 3000, 4000, and 5000 cm^2^/g are presented in Figure 10, Figure 11, Figure 12 and Figure 13. The graphs show that increasing the fineness of Portland clinker resulted in shortening the induction period, increasing the maximum heat release in the post-induction period, and increasing the heat of hydration after 72 h. Furthermore, there were more significant differences between cements made from clinkers with a specific surface area of 3000 and 4000 cm^2^/g than between 4000 and 5000 cm^2^/g; this was the result of the more significant relative difference between the smallest and average and average and largest specific surface areas.

The higher content of GGBFS in cement caused an increase in differences in the amount of hydration heat released between cements containing clinkers with a specific surface area of 4000 and 5000 cm^2^/g, compared to cements without it.

**Figure 14:**
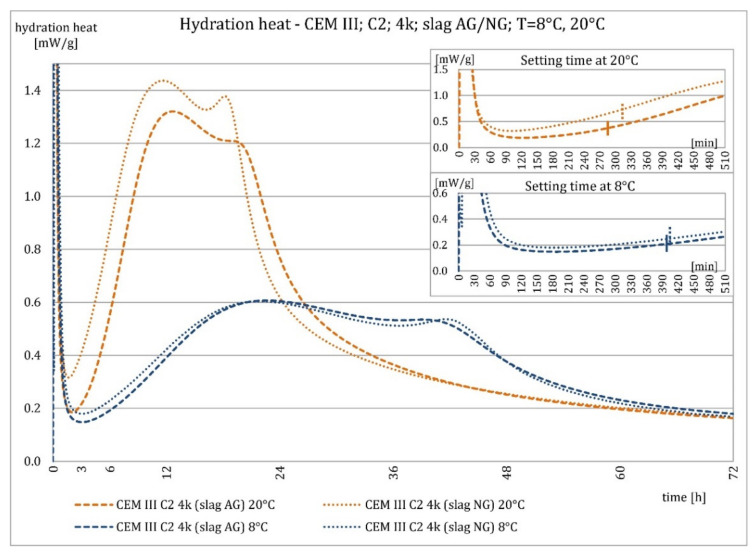
Hydration heat of C2 clinker based non-modified cement CEM III/A, containing slags differing in fineness at 8 °C and 20 °C.

**Figure 15:**
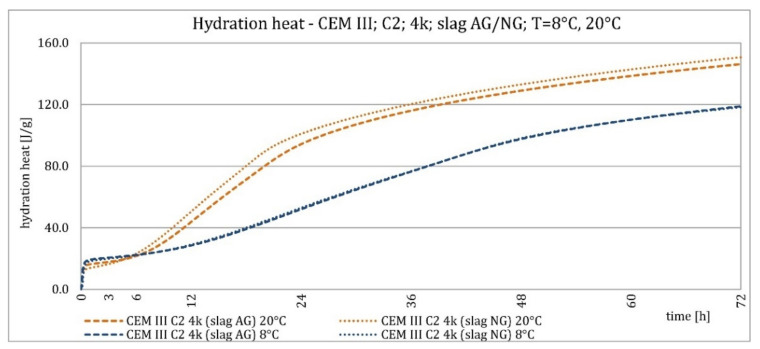
Hydration heat of C2 clinker based non-modified cement CEM III/A, containing slags differing in fineness at 8 °C and 20 °C.

Figure 14 and Figure 15 show the graphs of the heat of hydration of CEM III/A cement made from C2 clinker with a specific surface area of 4000 cm^2^/g and slag with specific surface areas of 3200 and 3870 cm^2^/g. The results were very similar. The plot shows that the use of slag with a lower specific surface area resulted in a slightly shorter induction period, a slightly higher maximum hydration heat release in the postinduction period, and a similar hydration heat after 72 h, compared to cement with slag treated with additional grinding.

**Figure 16:**
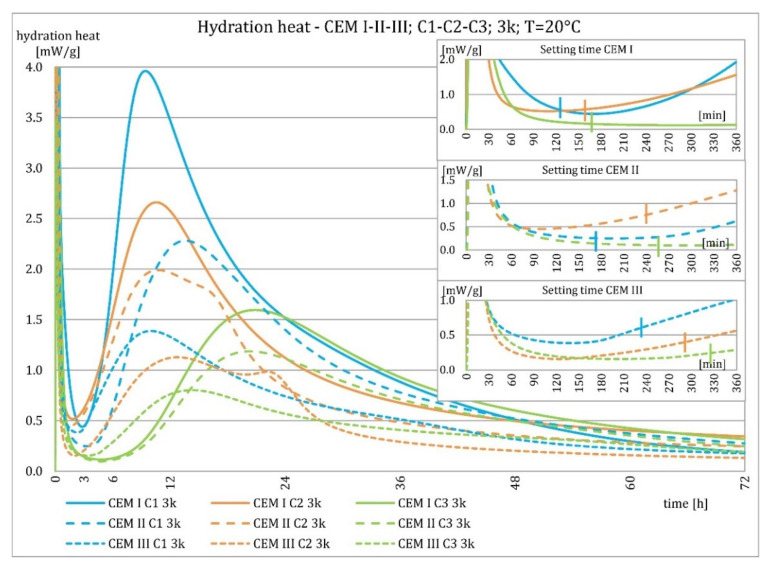
Hydration heat of C1-C2-C3 clinker based non-modified cements CEM I, CEM II/B-S, CEM III/A at 20 °C.

**Figure 17:**
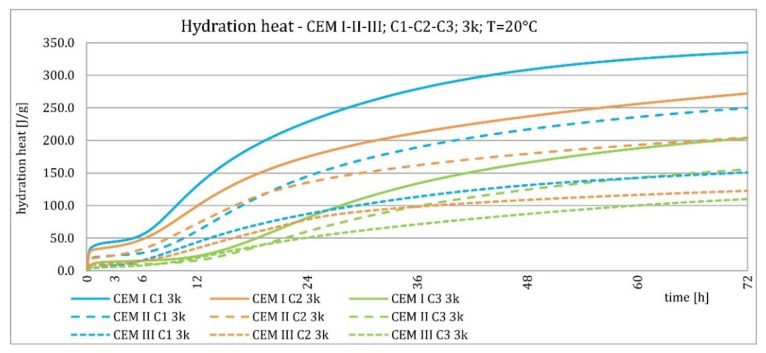
Hydration heat of C1-C2-C3 clinker based non-modified cements CEM I, CEM II/B-S, CEM III/A at 20 °C.

**Figure 18:**
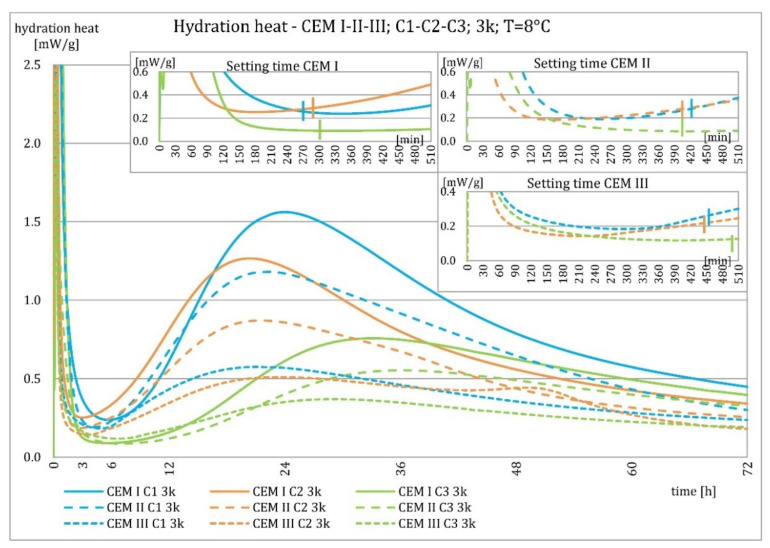
Hydration heat of C1-C2-C3 clinker based non-modified cements CEM I, CEM II/B-S, CEM III/A at 8 °C.

**Figure 19:**
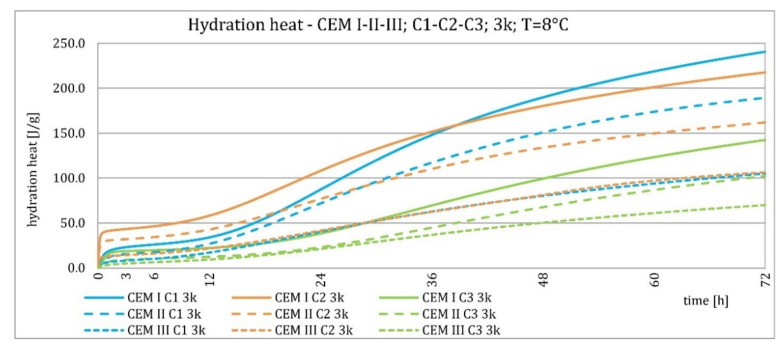
Hydration heat of C1-C2-C3 clinker based non-modified cements CEM I, CEM II/B-S, CEM III/A at 8 °C.

The evolution characteristics depended on the phase composition of the cement. These relationships are shown in Figure 16, Figure 17, Figure 18 and Figure 19. Cements made from clinker C1 had the highest maximum heat of hydration in the post-induction period and the highest heat released in 72 h. Cements made of C2 clinker were the second in order with respect to the above characteristics, and those made of C3 clinker were the last. It was valid for all types of cement, regardless of the amount of ground granulated blast furnace slag, although the differences were most evident for Portland cement CEM I. The heat of hydration evolution graphs for CEM III/A, made from C1 and C3 clinkers, did not reveal a second maximum peak in the post-induction period, which was the opposite of the C2 clinker.

As described earlier, the relationships occurring at an ambient temperature of 20 °C were also valid at 8 °C. Decreasing temperature caused an elongation of the induction period, an elongation of the postinduction period, a decrease in its maximum, and a decrease in the heat of hydration after 72 h. More evident than at higher temperatures was the relative differences in heat release between cements with different specific surface areas after 72 h. For all cements, it could be seen that up to about 36 h, the hydration heat was higher for cements made from C2 clinker compared to C1 clinker, which was much smaller at a higher temperature for cements CEM II/B-S and CEM III/A cements. Later, the relationship reversed.

**Figure 20:**
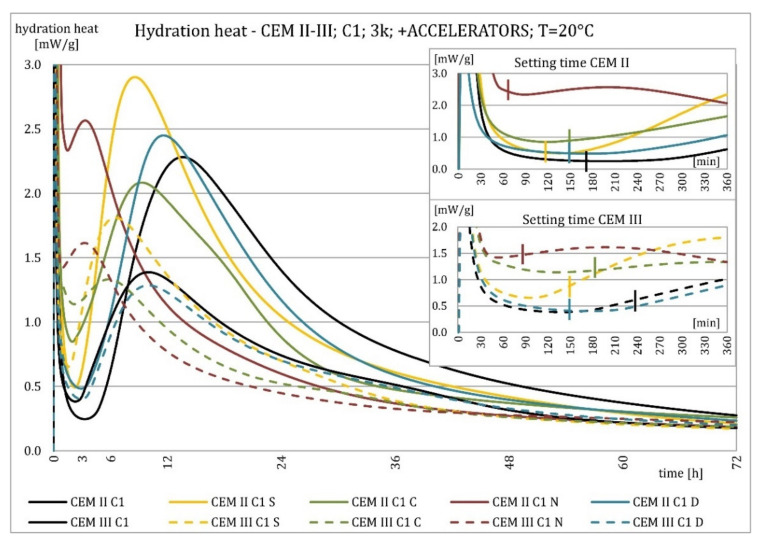
Hydration heat of C1 clinker-based cements CEM II/B-S, CEM III/A modified with accelerators at 20 °C.

**Figure 21:**
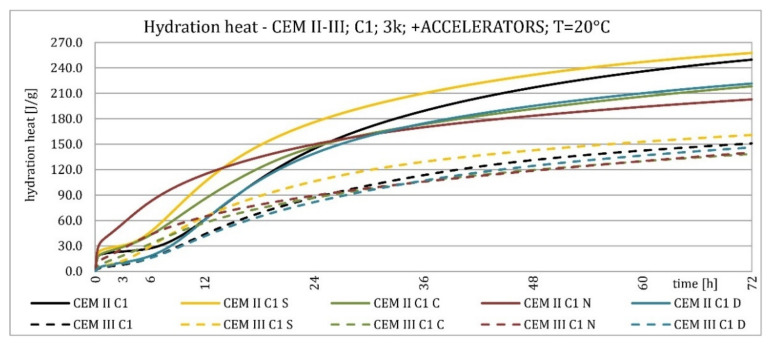
Hydration heat of C1 clinker-based cements CEM II/B-S, CEM III/A modified with accelerators at 20 °C.

**Figure 22:**
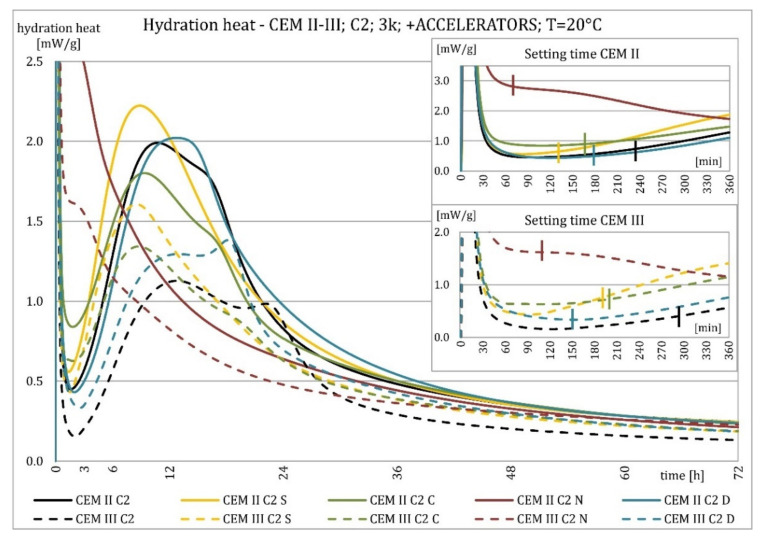
Hydration heat of C2 clinker-based cements CEM II/B-S, CEM III/A modified with accelerators at 20 °C.

**Figure 23:**
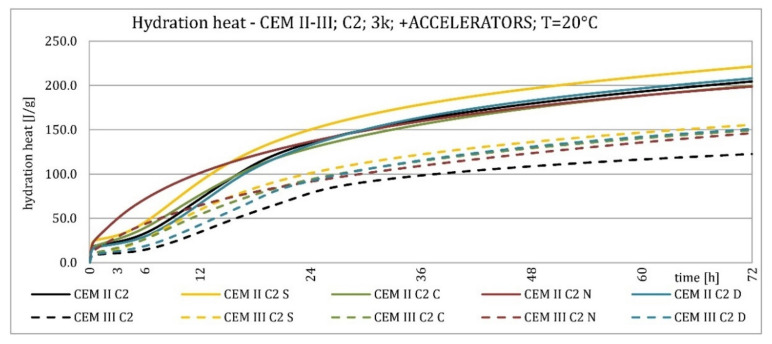
Hydration heat of C2 clinker-based cements CEM II/B-S, CEM III/A modified with accelerators at 20 °C.

**Figure 24:**
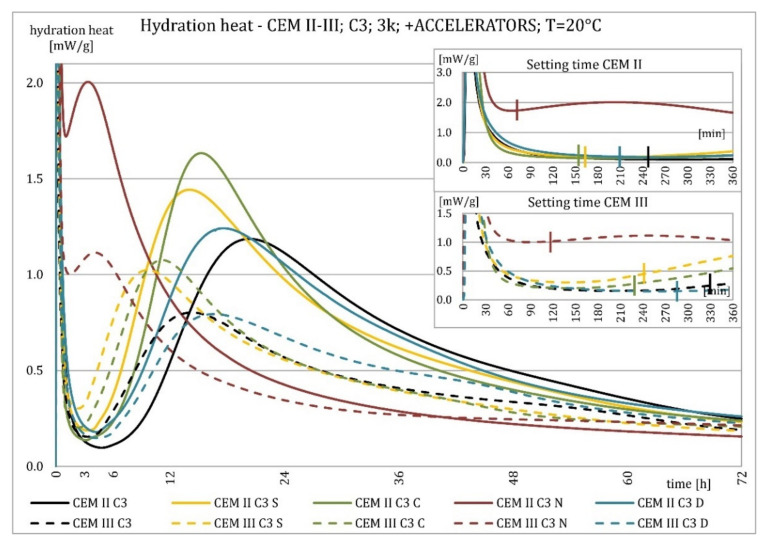
Hydration heat of C3 clinker-based cements CEM II/B-S, CEM III/A modified with accelerators at 20 °C.

**Figure 25:**
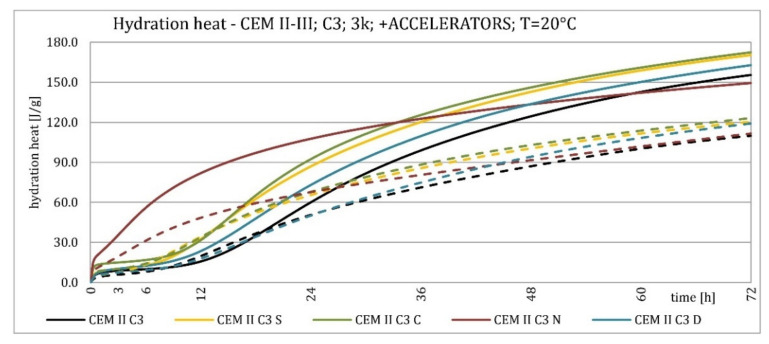
Hydration heat of C3 clinker-based cements CEM II/B-S, CEM III/A modified with accelerators at 20 °C.

**Figure 26:**
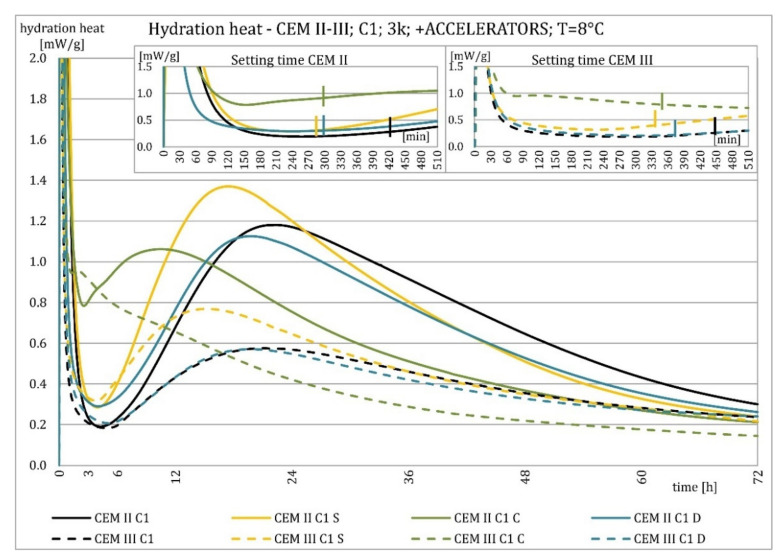
Hydration heat of C1 clinker-based cements CEM II/B-S, CEM III/A modified with accelerators at 8 °C.

**Figure 27:**
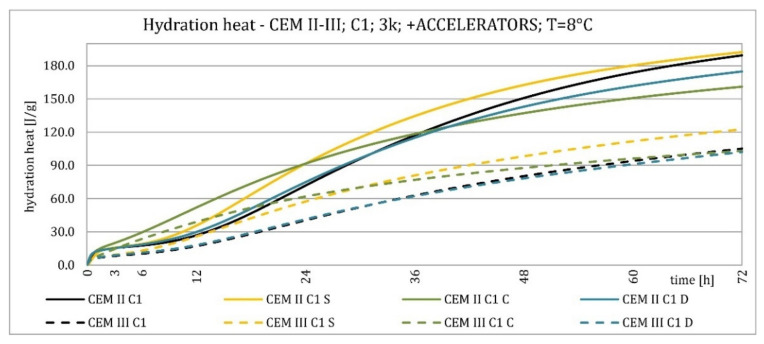
Hydration heat of C1 clinker-based cements CEM II/B-S, CEM III/A modified with accelerators at 8 °C.

**Figure 28:**
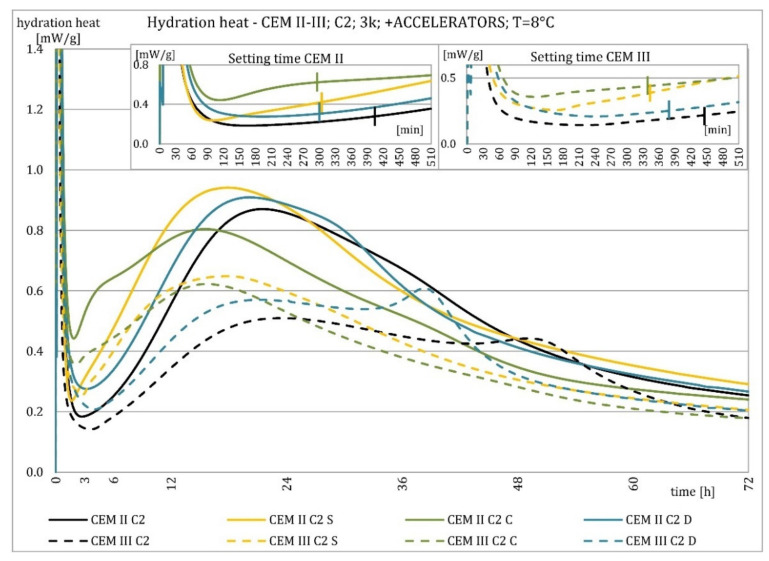
Hydration heat of C2 clinker-based cements CEM II/B-S, CEM III/A modified with accelerators at 8 °C.

**Figure 29:**
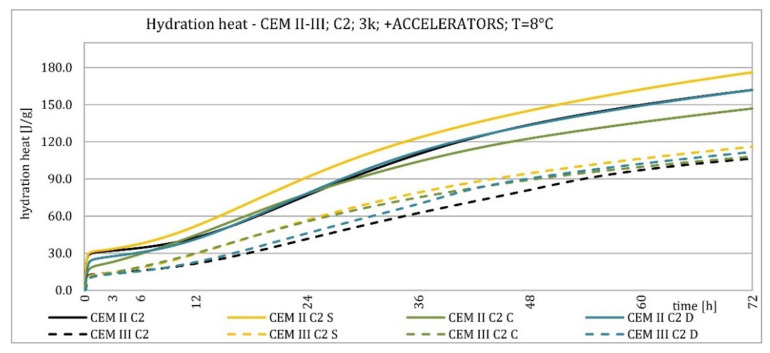
Hydration heat of C2 clinker-based cements CEM II/B-S, CEM III/A modified with accelerators at 8 °C.

**Figure 30:**
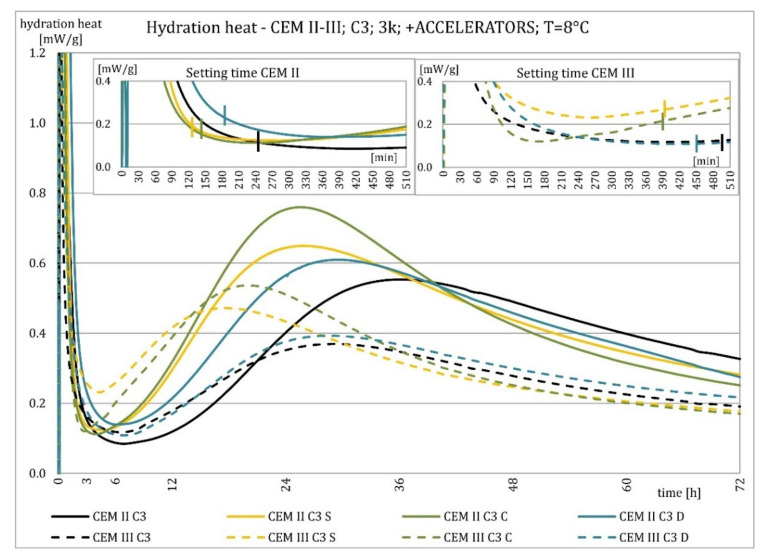
Hydration heat of C3 clinker-based cements CEM II/B-S, CEM III/A modified with accelerators at 8 °C.

**Figure 31:**
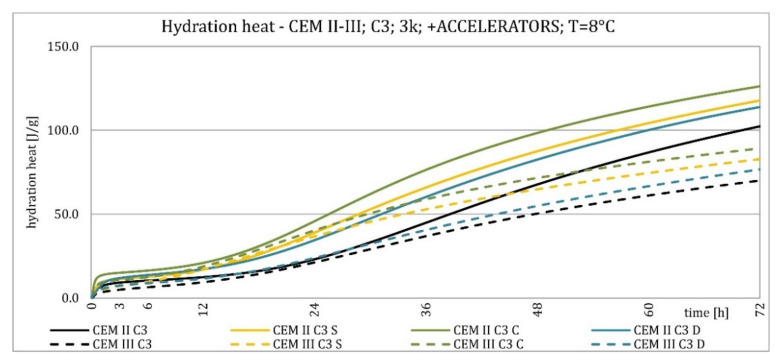
Hydration heat of C3 clinker-based cements CEM II/B-S, CEM III/A modified with accelerators at 8 °C.

The results of the hydration heat tests for accelerator-modified cements are presented in Figure 20, Figure 21, Figure 22, Figure 23, Figure 24, Figure 25, Figure 26, Figure 27, Figure 28, Figure 29, Figure 30 and Figure 31 for CEM II/B-S and CEM III/A, separately for cements containing different Portland clinkers. In the case of cements modified with admixture containing nucleation seeds (yellow curves), the induction period was shortened, the maximum hydration heat evolved in the post-induction period increased and occurred earlier, and the amount of hydration heat evolved after 72 h was increased. The accelerator was slightly more effective in CEM III/A than in CEM II/B-S and cement made from clinkers containing less C_3_A (C2 and C3).

The effect of calcium nitrate (green curves) differed to a greater extent depending on the type of clinker and the content of ground granulated blast furnace slag in the cement. In the case of CEM II/B-S cement, it was most effective in cooperation with cement made from C3 clinker, which contained the most C_2_S. It increased the most in the post-induction period and heat release after 72 h. In cooperation with the other two clinkers, the maximum heat released decreased, and the heat after 72 h remained unchanged. In the case of all cements, it shortened the induction period, and in cements made of C2 and C3 clinkers, the maximum heat release occurred earlier. Calcium nitrate had a similar performance for CEM III/A cements made from clinkers C2 and C3. There was an increase in the maximum heat release in the post-induction period and the heat released after 72 h. In association with clinker C1, the heat of hydration after 72 h was lower and the maximum heat release in the post-induction period did not change compared to the reference sample.

Sodium hydroxide (burgundy curves) greatly shortened the initial setting time and completely changed the characteristics of the calorimetric curve. In the case of CEM II/B-S and CEM III/A cements made from C1 clinker, the induction period could not be distinguished. In the other types of cement, this period was concise and characterized by a much higher amount of heat released than when modified with the other activators. A higher heat of hydration characterizes the CEM II/B-S cement after 72 h. After 72 h of CEM III/A cement, the heat of hydration did not increase.

The calorimetric curves of the CKD-modified cements (blue curves) were similar to that for the reference samples (black curves), regardless of clinker type and the content of GGBFS. No clear correlation of the effectiveness of dust as an activator with the phase composition of the clinker could be seen. The maximum heat release in the post-induction period did not decrease in any of the cases. In addition, no elongation of the induction period was recorded. In cases where it was shortened, it was of a small magnitude. A slight decrease in the heat of hydration was observed after 72 h only in the case of cement made of C1 clinker. CKD showed slightly higher efficiency in CEM II/B-S cement, which was visible in the earlier occurrence of the maximum release of the heat of hydration. This change was not visible in the case of cements with a higher GGBFS content. In this research, the beneficial influence of CKD was observed; however, it is necessary to recall the different actions of various CKD [49].

### 3.3. Summary

Summaries of accelerator effects on hydration heat and initial setting time are presented in Table 8 for 20 °C and 8 °C. All accelerators shortened the initial setting time. The duration of the dormant period was shortened in almost all cases. Only CKD modification did not result in a change in duration. The maximum value of heat in the post-dormant period (second peak) was always greater with accelerator usage. The only exception was calcium nitrate in cooperation with CEM II/B-S based on clinker C1 and C2. It appeared to be caused by the potential overdose of calcium nitrate in ratio to clinker. It was not visible for CEM III/A because of the too high GGBFS content. All accelerators, except CKD, caused an earlier occurrence of the second peak. A similar exception was for CEM II/B-S based on calcium nitrate C1 and C2 clinker. The total heat evolved after 72 h was lower, higher, or similar in various cases. There was no rule behind it.

## 4. Discussion

### 4.1. Initial Setting Time

The initial setting time depends on the content of tricalcium aluminate (C_3_A) in the cement [32,69]. As its amount increases, the amount of ettringite formed increases, which causes a faster build-up of the spatial crystalline structure [70,71]. Increasing the specific surface area of the clinker shortens the initial setting time, the larger the cement grain area available for the hydration reaction. The initial setting time at the lower temperature is not significantly different using the different clinkers for Portland slag cement and blast furnace cement. These various behaviors of blended cements at different temperatures should be the subject of more detailed research. Increasing the proportion of GGBFS, a nearly inert component in the initial stage of hydration [72,73], increases the amount of SO_3_ per unit mass of C_3_A derived from the clinker. Consequently, in cement containing clinker with a higher amount of this phase, the hydration reaction is proportionally faster than in Portland cement. Increasing the content of GGBFS causes an increase in the distance between the clinker grains, which have to be filled with hydration products. A smaller particle size results in an increase in the surface area of the grains available for the reaction to occur, and the reaction rate is faster [2,32].

The explanation for accelerators efficiency varies depending on their chemical nature and physical properties. In the case of sodium hydroxide, which acts mainly on the C_2_S and C_3_S phases, rapid cement setting occurs [3,74]. Its cooperation with cement is similar due to the similar sum content of these phases. A similar explanation exists for the use of calcium nitrate, which increases efficiency as the content of these two phases, especially belite, increases [7]. This was particularly evident in the case of the C3 clinker (18.2% C_2_S), for which it was the second most effective accelerator. Nucleation seeds-containing admixture introduces additional microparticles into the batch water. As a result, it serves as initial crystallization points. Therefore, hydration products can not only be on the surface of cement grains [64]. CKDs that also contain alkalis and sulfides that serve as slag activators can act similarly [2,3,72]. As a result of activators containing nucleation seeds and calcium nitrate, it was possible to bridge the difference between the CEM-CEM II and CEM II-CEM III cement pairs. However, CKD did not work as well with all of them.

### 4.2. Hydration Heat

Cements made with C1 clinker were characterized by the highest heat released during the first 72 h and the highest maximum hydration heat released during the post-induction period. Cements containing C2 clinker were second, and those with C3 clinker were third. This observation was independent on the content of ground granulated blast furnace slag. Increasing the specific surface area of the clinker resulted in a shortening of the induction period, an increase in the maximum heat release in the post-induction period, and an increase in the heat released after 72 h, which is confirmed in the literature [2,32]. The higher GGBFS ratio caused greater differences in hydration heat for cements of different specific surface areas. It can be explained by averaging the specific surface area by using slag with the same specific surface area in all cements in increasing amounts. Furthermore, the plot for CEM III/A cement showed a characteristic second maximum occurring in the post-induction period.

The development of hydration heat release was caused by differences in the content of the C_3_A and C_3_S phases in clinkers. Clinker C1 contained the most considerable amount of the C_3_A phase—13.5% and an average C_3_S content—64.4%. Clinkers C2 and C3 contained a similar amount of C_3_A—4.0% and 2.4%, respectively, but differed significantly in the content of C_3_S (68.7% and 58.0%, respectively)—the phase in which hydration was the second-largest source of heat released during the reaction of cement with water [2,32].

Crystallization seeds’ action in cements made from different clinkers was similar due to the specific nature of this activator and the introduction of additional nuclei of crystallization around which the formation of hydration products can begin. Calcium nitrate worked better in cements with higher GGBFS content and in cements made from clinkers with a lower C_3_A content and a higher C_2_S content. For CEM III/A manufactured with clinkers C1, C2, calcium nitrate changed the course of the heat release graph. It might be caused by the overdosage of this admixture [3,7,11]. However, no correlation could be seen between the effectiveness of this activator and the content of the C_3_S phase in which it should also act, considering the data in the literature [3,7]. Sodium hydroxide completely changed the hydration process. Therefore, it was not used at 8 °C. In this research, the beneficial influence of CKD was observed by the small amount of dust added and its important specific surface area. However, it is necessary to recall the different actions of various CKDs [49].

## 5. Conclusions

The main contribution of this paper is to assess and compare different possibilities of cement setting acceleration.

Factors that shorten the setting time can be ranked. The most effective is to increase the temperature of the ingredients and the environment; the second is to reduce the GGBFS content in cement and use accelerators. The least effective is the additional grinding of the Portland clinker.

To accelerate cement setting, in most cases, additional clinker grinding, which is a very energy-intensive process [75], can be successfully replaced by the use of setting accelerators. Accelerators were not sufficient to compensate for the temperature difference.

Setting activators worked better with cement with lower GGBFS content, leading to the conclusion that these activators affect mainly Portland clinker, at least in the initial phase of cement hydration. The efficiency depended on the phase composition of the Portland clinker.

The effect of changing the characteristics of the evolution of the hydration heat using nucleation seeds was similar, irrespective of the cement. However, the effectiveness seemed slightly higher for cement that contained less C_3_A. The effectiveness of calcium nitrate depended on the type of clinker and the GGBFS content in the cement. This research focused on the hydration heat, but more research on amounts of the C-S-H phase and portlandite should be conducted to assess the hydration rate. XRD and TGA-DTG methods for different ages of blended cement pastes may be involved.

## Figures and Tables

**Table 1:** Chemical composition of GGBFS, mass %.

SiO_2_	Al_2_O_3_	Fe_2_O_3_	CaO	MgO	SO_3_	Cl^−^	Na_2_O	K_2_O	Ign. Loss.	Blaine’s Specific Surface Area
37.35	7.30	1.22	43.90	5.73	0.62	0.03	0.55	0.56	0.17	Symbol NG: 3200 cm^2^/g Symbol AG: 3870 cm^2^/g

**Table 2:** Phase composition of Portland clinkers, mass %.

Symbol	C_2_S	C_3_S	C_3_A	C_4_AF
C1	10.9	64.4	13.5	7.3
C2	12.4	68.7	4.0	12.1
C3	18.3	58.0	2.4	17.5

**Table 3:** Chemical composition of Portland clinkers, mass %.

Sym.	SiO_2_	Al_2_O_3_	Fe_2_O_3_	CaO	MgO	SO_3_	Cl^−^	Na_2_O	K_2_O	CaO_free_	Ignition Losses	Blaine’s Specific Surface Area
C1	20.25	6.83	3.23	65.66	1.39	0.69	0.01	0.15	1.02	2.80	0.15	3000; 4000; 5000 cm^2^/g
C2	21.25	5.00	3.40	64.81	2.09	0.55	0.03	0.11	1.03	1.48	0.35	3000; 4000; 5000 cm^2^/g
C3	21.62	4.58	5.75	65.38	0.79	0.27	0.01	0.30	0.25	0.95	0.25	3000; 4000; 5000 cm^2^/g

**Table 4:** Chemical composition of anhydrite, mass %.

SiO_2_	CaO	MgO	SO_3_	Na_2_O	Ignition Losses
0.61	40.16	0.40	54.83	0.02	2.71

**Table 5:** Chemical composition of CKD, mass %.

SiO_2_	Al_2_O_3_	Fe_2_O_3_	CaO	MgO	SO_3_	Cl^−^	Na_2_O	K_2_O	P_2_O_5_	TiO_2_	Mn_2_O_3_	SrO	ZnO	Ignition Losses
18.16	4.55	2.03	57.13	1.26	1.95	1.44	0.12	2.38	0.12	0.22	0.06	0.10	0.24	11.70

**Table 6:** Composition of cement pastes for initial setting time tests.

Cement Type	Portland Clinker		GGBFS	Anhydrite	Water	CKD	Admixture		
	type	[g]	[g]	[g]		[g]	type	[% m.c.]	[g]
CEM I	C1	439.1	-	10.9	Amount of water depends on cement type and fineness, accelerator used and temperature during the test. Results given in Table 7.	-	-	-	-
	C2	438.0	-	12.0		-	-	-	-
	C3	435.7	-	14.3		-	-	-	-
CEM II	C1	285.3	153.6	11.07		-	-	-	-
							S	4.0	18.0
							C	2.0	9.0
							N	5.0	22.5
		256.8	138.2	10.0		45.0	D	-	-
	C2	284.8	153.4	11.8		-	-	-	-
							S	4.0	18.0
							C	2.0	9.0
							N	5.0	22.5
		256.3	138.0	10.7		45.0	D	-	-
	C3	283.9	152.9	13.3			-	-	-
							S	4.0	18.0
							C	2.0	9.0
							N	5.0	22.5
		255.5	137.6	12.0		45.0	D	-	-
CEM III	C1	153.6	285.2	11.3		-	-	-	-
							S	4.0	18.0
							C	2.0	9.0
							N	5.0	22.5
		138.2	256.7	10.1		45.0	D	-	-
	C2	153.4	284.9	11.7		-	-	-	-
							S	4.0	18.0
							C	2.0	9.0
							N	2.0	22.5
		138.1	256.4	10.5		45.0	D	-	-
	C3	153.1	284.4	12.5			-	-	-
							S	4.0	18.0
							C	2.0	9.0
							N	5.0	22.5
		137.8	256.0	11.2		45.0	D	-	-

**Table 7:** Water demand depending on cement type, accelerator used, and temperature.

	**Water Amount [g]**					
Clinker	C1	C2	C3	C1	C2	C3
Temperature	20 °C			8 °C		
Symbol of cement						
CEM I C * 3k **	193	147	120	188	139	112
CEM I Cx4k	197	168	121	186	160	115
CEM I Cx5k	203	172	129	191	162	119
CEM II Cx3k	195	134	116	190	124	111
CEM II Cx4k	197	138	118	189	128	106
CEM II Cx5k	198	148	124	192	136	113
CEM III C× 3k	135	135	122	128	129	113
CEM III Cx 4k	136	137	122	124	131	117
CEM III Cx 4k NG ***	135	135	122	122	128	117
CEM III Cx 5k	146	143	124	137	132	117
CEM II Cx 3k S ****	196	135	110	190	125	103
CEM II Cx 3k C	152	135	125	146	126	116
CEM II Cx 3k N	181	167	142	not tested at 8 °C		
CEM II Cx 3k D	146	133	119	140	125	110
CEM III Cx 3k S	129	123	114	119	116	106
CEM III Cx 3k C	135	130	128	126	123	120
CEM III Cx 3k N	158	145	144	not tested at 8 °C		
CEM III Cx 3k D	133	130	123	123	118	117

* all types of cement were prepared from all clinkers; x denotes which. ** 3k, 4k, 5k indicates Blaine’s specific surface area of cement—3000, 4000, 5000 cm^2^/g. *** NG—typically ground slag to Blaine’s specific surface area 3200 cm^2^/g. The remaining mixtures were prepared with AG—additionally ground slag of Blaine’s specific surface area 3870 cm^2^/g. **** S, C, N, D—symbols of accelerators.

**Table 8:** Effects of accelerators on hydration heat and initial setting time in comparison to non-modified cements.

Temperature	Clinker	Cement	Accelerator	Influence On				
				Initial Setting Time	Dormant Period Length	Maximum Value in Post-Dormant Period	Time of Maximum Occurrence	Heat after 72 h
20 °C	C1	II/B-S	S	-	-	+	←	+
			C	-	-	-	←	0
			N	-	-	+	←	-
			D	-	-	+	←	0
		III/A	S	-	-	+	←	+
			C	-	-	0	←	-
			N	-	-	+	←	-
			D	-	0	0	0	-
	C2	II/B-S	S	-	-	+	←	+
			C	-	-	-	0	0
			N	-	-	+	←	+
			D	-	0	0	0	0
		III/A	S	-	-	+	←	+
			C	-	-	+	←	+
			N	-	-	+	←	+
			D	-	0	+	0	+
	C3	II/B-S	S	-	-	+	←	+
			C	-	-	+	←	+
			N	-	-	+	←	-
			D	-	-	0	←	+
		III/A	S	-	-	+	←	+
			C	-	-	+	←	+
			N	-	-	+	←	-
			D	-	0	0	0	+
8 °C	C1	II/B-S	S	-	-	+	←	0
			C	-	-	0	←	-
			D	-	0	0	0	-
		III/A	S	-	-	+	←	+
			C	-	-	+	←	0
			D	-	0	0	0	0
	C2	II/B-S	S	-	-	+	←	+
			C	-	-	0	←	-
			D	-	0	0	0	0
		III/A	S	-	-	+	←	+
			C	-	-	+	←	0
			D	-	0	+	←	0
	C3	II/B-S	S	-	-	+	←	+
			C	-	-	+	←	+
			D	-	0	+	←	+
		III/A	S	-	-	+	←	+
			C	-	-	+	←	+
			D	-	0	0	0	+

-—Shortening of initial setting time; shortening of the dormant period; lower maximum in the post-dormant period, lower heat after 72 h. +—Higher maximum in the post-dormant period, higher heat after 72 h. 0—No changes. ←—Earlier occurrence of maximum in the post-dormant period.

## Data Availability

Not applicable.
